# Validation of automated script-based proton therapy optimisation for comparative head and neck planning

**DOI:** 10.1016/j.phro.2026.101058

**Published:** 2026-08-05

**Authors:** Marte Kåstad Høiskar, Ulrik Vindelev Elstrøm, Peter Sandegaard Skyt, Kathrine Røe Redalen, Sigrun Saur Almberg

**Affiliations:** a Department of Physics, Norwegian University of Science and Technology, Trondheim, Norway; b Danish Centre for Particle Therapy, Aarhus University Hospital, Denmark; c Department of Radiotherapy, Cancer Clinic, St. Olavs Hospital, Trondheim University Hospital, Trondheim, Norway

**Keywords:** Head and neck cancer, Proton therapy, Automated optimisation, Comparative planning

## Abstract

**Background and purpose:**

Proton and photon plan comparison is often the gateway to proton therapy for head and neck cancer (HNC). Manual planning is resource demanding, whereas automated planning may efficiently create consistent treatment plans. An automated rule-based intensity-modulated proton therapy (IMPT) plan optimisation script was developed within a commercial treatment planning system for comparative planning. This study benchmarked automated against clinical plans and evaluated whether a standard or patient-specific field set-up was necessary.

**Materials and methods:**

Twenty HNC patients previously treated with IMPT using manual plans were included. For each patient, two automated IMPT plans were generated, differing in field set-up: one with a standard five-field set-up and one matching the clinical field set-up. Robustness, dose distribution, and normal tissue complication probability (NTCP) for dysphagia and xerostomia were compared.

**Results:**

On average, automated IMPT plans were created in 64 min. Compared with clinical plans, both automated plan types, generally, reduced organ of interest mean doses, with median differences of up to 3.3 Gy (glottis). Automated plans also had lower median NTCP for dysphagia of 0.1%-points (interquartile range (IQR): −0.8–0.9) and 0.3%-points (IQR: 0.0–0.6) for standard and clinical field set-ups, respectively. For xerostomia, the corresponding results were 0.3%-points (IQR: 0.0–1.5) and 0.3%-points (IQR: 0.1–0.8), respectively.

**Conclusions:**

The IMPT plan optimisation script created plans with comparable robustness, dose distribution, and NTCP to clinical plans and is applicable for selecting HNC patients for IMPT. Additionally, a standard field set-up was adequate for comparative planning for most patients.

## Highlights

• Automated optimisation script created proton plans in less than 1.5 h.
• Automated plans were benchmarked against clinical plans for head and neck cancer.
• Median difference in mean doses to organs of interest was <3.3 Gy.
• Difference in median normal tissue complication probability was <0.3%-points.
• Standard field set-up was adequate for comparative planning for most patients.

## Graphical abstract

**Figure:**
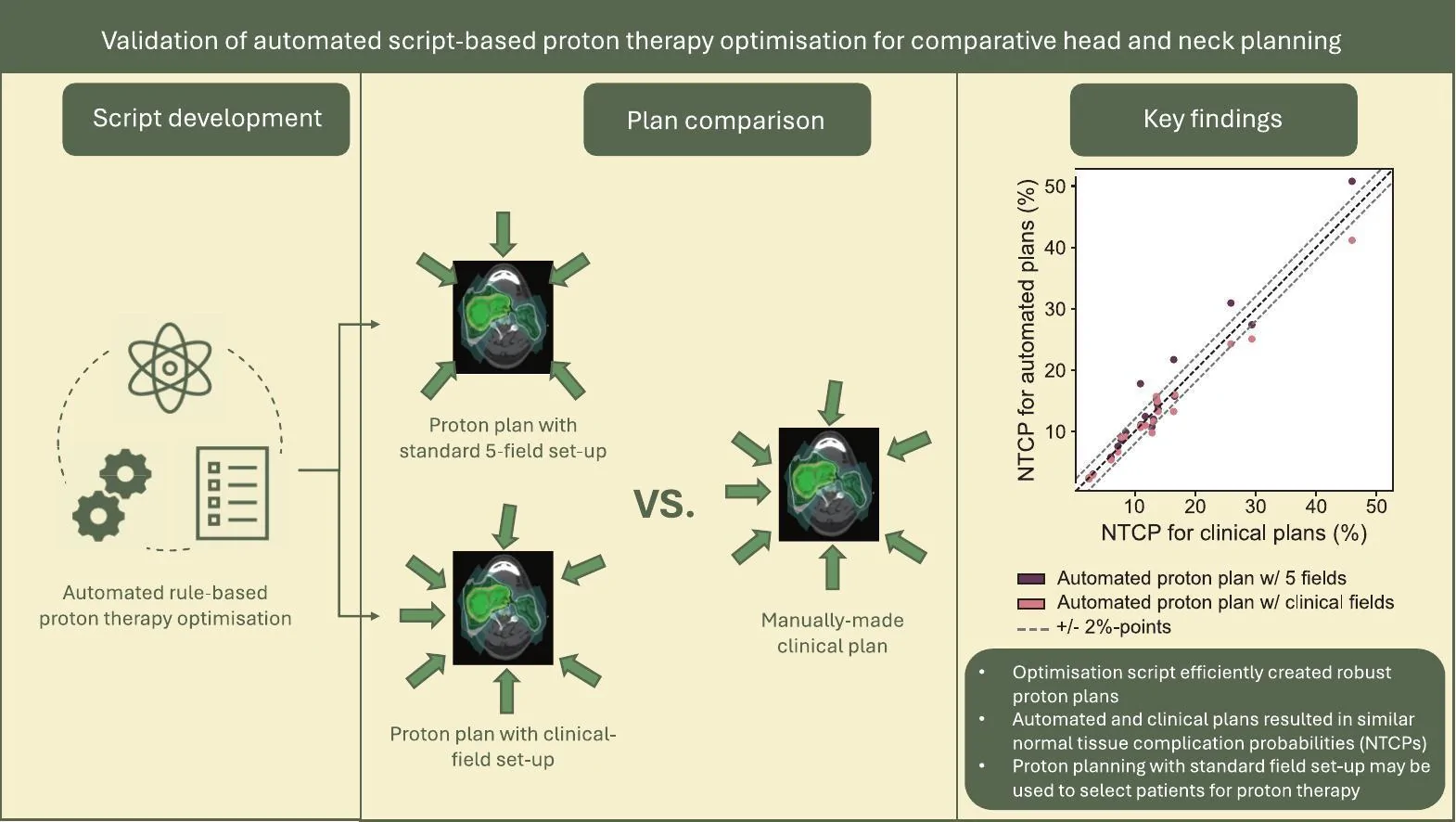

## 1 Introduction

Ongoing studies are investigating potential benefits of proton therapy for head and neck cancer (HNC) [1], [2], [3], [4], although, photon therapy is still regarded as standard treatment. In some countries, the choice of radiation modality depends on the estimated risk of side effects using model-based approaches [5], [6], [7]. For each patient, comparative planning is performed, meaning that photon and proton plans are generated and compared before a treatment decision is made. Proton therapy is chosen if it reduces the normal tissue complication probability (NTCP) for side effects, e.g. dysphagia and xerostomia. Intensity modulated proton therapy (IMPT) plan optimisation is time and resource demanding [8]. Automated IMPT plan optimisation may efficiently create consistent treatment plans independent of planner experience [9], [10].

Several automated IMPT plan optimisation methods have been developed, either data-driven [9], [11], [12] or rule-based [10], [13]. Data-driven IMPT plan optimisation can make high-quality plans within ten minutes [11]; however, it requires large, well-curated training datasets. In contrast, rule-based optimisation requires no training data and only needs to update its rules when protocols change. Therefore, rule-based IMPT plan optimisation scripts are easier to implement and maintain in the clinics. Kong et al. have developed a promising fully automated rule-based, robust multi-criteria optimisation method [10], [14]. However, it uses an in-house treatment planning system (TPS) and requires up to four hours per plan.

Although increasing the number of fields decreases organ of interest (OOI) doses for IMPT [13], it also increases treatment delivery time. Therefore 4–6 fields are often used clinically for HNC IMPT. Patient-specific adjustments to field set-ups may improve plan quality but introduce inter-planner variability. A standard field set-up may be sufficient and help automate IMPT plan optimisation for consistent comparative planning.

We propose a fast rule-based automated IMPT plan optimisation script developed for a commercial TPS. Script-based IMPT plans were compared with manually created and clinically approved IMPT plans for HNC. The aim was to validate the script and investigate whether a standard field set-up is sufficient for efficient comparative treatment planning.

## 2 Methods

### 2.1 Patient population

This retrospective study included 20 HNC patients treated consecutively with IMPT at the Danish Centre for Particle Therapy (DCPT). Patients were eligible for IMPT if the NTCP for grade 2 dysphagia and/or grade 3 xerostomia was reduced by >5%-points compared to volumetric modulated arc therapy (VMAT). All patients had oropharyngeal cancer, except two with laryngeal cancer. No interventions were required. Permission to use the patient data was granted by local hospital authorities.

### 2.2 Treatment planning

For each patient, four treatment plans were made: one VMAT and three IMPT plans, prescribing median doses of 66 Gy (33 fractions) or 68 Gy (34 fractions) to the clinical target volume (CTV), denoted as CTV_66/68Gy_. CTV_60Gy_, defined as the CTV_66/68Gy_-volume expanded by 5 mm, was prescribed 60 Gy, while elective lymph nodes (CTV_50Gy_) were prescribed 50 Gy. All plans followed the Danish Head and Neck Cancer Group (DAHANCA) guidelines [15], aiming to minimise OOI doses while maintaining robust CTV coverage.

Among the three IMPT plans, one clinical plan was created manually in Eclipse v16.1.0 (Varian Medical Systems, Palo Alto, California, USA) and two plans were created automatically in RayStation v2023B (RaySearch Laboratories AB, Stockholm, Sweden). All IMPT plans used a fixed relative biological effect of 1.1 during optimisation and were initially based on a standard five-field set-up (0°, 50°, 160°, 200° and 310°). Anterior and lateral posterior fields only irradiated inferior of the chin and superior of the shoulder, respectively. Lateral anterior and lateral posterior fields irradiated target ipsilaterally. However, posterior fields were allowed to irradiate contralaterally at parotid level to spare parotids. Clinical plans had a superior border for posterior fields to spare cerebellum; this was not implemented for automated plans. They were also individualised by adjusting field angles and/or adding more fields, resulting in 5–7 fields in total. A posterior field, irradiating the chin- and shoulder region, was added for 50% of the clinical plans to ensure all parts of target were covered by at least two fields. For clinical plans, the mean dose for CTV_66/68Gy_ was normalised.

The two automated IMPT plan types differed in field set-up: one utilised the standard five-field set-up, the other mimicked the field set-up of clinical plans. Clinical field set-ups often included a posterior field. The corresponding automated plans restricted spot placements to the shoulder- and chin-region when the shoulder and chin were in proximity in the longitudinal direction; otherwise, no restrictions were applied to the posterior field. All IMPT plans used a 3 cm range shifter, except two clinical IMPT plans (5 cm).

Clinical planning calculated dose with the Proton Convolution Superposition algorithm (Eclipse) with 1.5 mm^3^ voxels, whereas automated planning used Monte Carlo (RayStation) and 2 mm^3^ voxels. All plans were robustly optimised with a combined isotropic position uncertainty of 4 mm and a density uncertainty of ±3.5%. To evaluate IMPT plan robustness, 14 scenarios were calculated by combining density uncertainty with position uncertainty. Automated and clinical IMPT plans were considered robust if the worst-case scenario had V_95%_ > 99% for CTV_66/68Gy_, V_95%_ > 98% for CTV_50Gy_ and CTV_60Gy_, D_max_ < 50 Gy for spinal cord and D_max_ < 60 Gy for brainstem. To assess hotspots, the nominal scenario required V_107%_ < 1.8 cm^3^ for the body. V_x_ refers to the volume receiving x% of the prescribed dose. The maximum dose (D_max_) was the minimum dose delivered to the volume of 0.03 cm^3^ receiving the highest dose within the structure.

VMAT plans were made in RayStation (v2024B) using an optimisation script that is developed and in clinical use at St. Olavs Hospital [16]. The script prescribed dose to the planning target volumes (PTVs) to ensure robust target coverage. PTV_60Gy_ and PTV_66/68Gy_ were the CTV_60Gy_ and CTV_66/68Gy_ respectively, expanded by 4 mm, while PTV_50Gy_ was the CTV_50Gy_ expanded by 5 mm. PTV_50Gy_, PTV_60Gy_, and PTV_66/68Gy_ were prescribed 50 Gy, 60 Gy and 66/68 Gy, respectively. VMAT plans consisted of a single 6 MV arc using a sliding window technique with an Elekta Agility linear accelerator. Dose was calculated using the collapsed cone convolution algorithm with 2 mm^3^ voxels.

### 2.3 Automated IMPT plan optimisation script

The automated IMPT plan optimisation script was inspired by the VMAT optimisation script [16]. It is an in-house Python (v3.11) script developed within the RayStation scripting environment. Automated IMPT plans were made on a computer with an AMD Ryzen Threadripper PRO 5945WX 12-cores CPU and an NVIDIA RTX A6000 GPU.

**Fig. 1:**
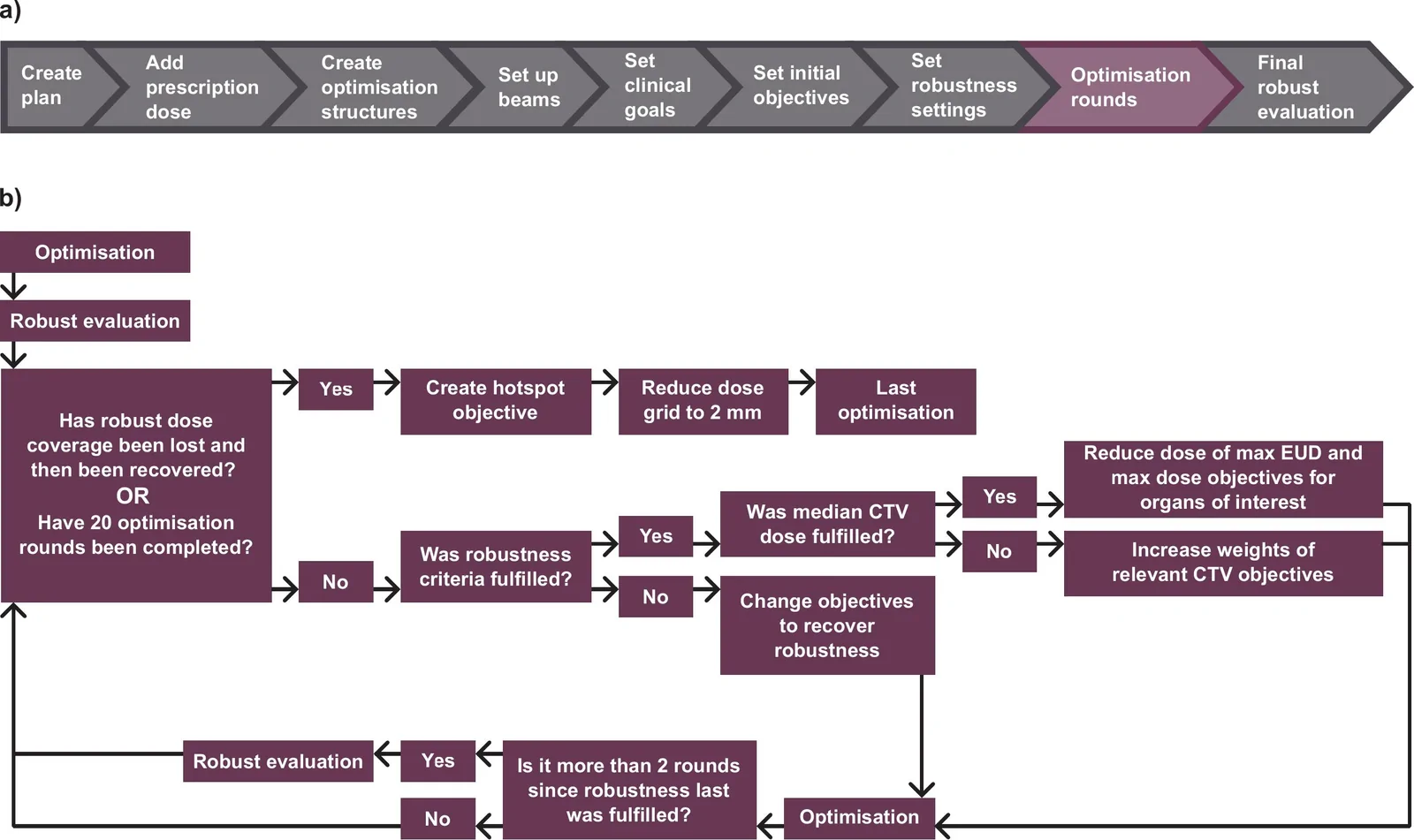
a) Flowchart of the script-based automated intensity modulated proton therapy planning process, which includes several rounds of dose optimisation. b) A more detailed flowchart of the dose optimisation. CTV; clinical target volume. EUD; equivalent uniform dose.

The steps performed by the script are described in Fig. 1a. An initial IMPT plan with user-determined prescription, script-generated optimisation structures, a standard field set-up, predefined clinical goals, initial objectives and robustness settings was created. The script paused before optimising the dose distribution, allowing users to adjust the initial plan. When creating automated plans with clinical field set-up, only the field set-up was changed to mimic clinical plans. Subsequently, the script performed iterative optimisations with intermediate robust evaluations (Fig. 1b). The first optimisation used the initial objectives to run 100 iterations. Further optimisations, with 60 iterations each, were then performed until the stopping criteria were met; either robust CTV coverage was lost after the first four optimisations and subsequently regained, or a maximum of 20 optimisations was completed. The number of iterations increased to 100 whenever robust CTV coverage was lost. Lastly, an objective to remove hotspots was added, and the final dose was optimised with 2 mm instead of 3 mm resolution before a final robust evaluation.

### 2.4 Plan comparison

The biological weighted mean dose (D_mean_) and D_max_ of OOIs, and median dose (D_50%_) of CTVs were compared. For all OOIs and CTVs, a Wilcoxon signed-rank test was performed to test the null hypothesis that differences in D_mean_, D_max_ or D_50%_ between automated and clinical plans were zero. Experienced proton physicists qualitatively evaluated dose distributions.

NTCP of dysphagia (grade 2) and xerostomia (grade 3) was calculated for each plan using models applied by DAHANCA [15], which are based on Dutch models [17] but have been adapted to reflect the Danish patient population [7]. The NTCP model for dysphagia included mean doses for the oral cavity and pharyngeal constrictor muscles (PCMs), whereas the model for xerostomia included mean doses for the submandibular and parotid glands. ΔNTCP was the NTCP difference between VMAT and IMPT plans (VMAT-IMPT). Paired differences in NTCP and ΔNTCP between automated and clinical IMPT plans were evaluated with a Wilcoxon signed-rank test. Bonferroni correction was applied to correct for multiple testing, resulting in a 0.1% significance level.

## 3 Results

The initial IMPT plan required the planner's attention and took the script approximately 20 min to create. For the next step, i.e. the fully automated optimisation, median (interquartile range) time was 64 (57–83) min and 64 (54–78) min for plans with a standard five-field and a clinical field set-up, respectively. Experienced proton physicists required approximately 2–3 days to create a clinical plan.

All clinical and automated IMPT plans with clinical field set-ups fulfilled all robustness criteria. Among the automated IMPT plans with a standard five-field set-up, six (30%) failed the robustness requirements. Four (20%) of these failed the requirement for maximum body dose; three also failed to achieve robust CTV coverage. Two (10%) other plans did not achieve robust CTV coverage in one scenario. Additional information about the CTV coverage of the six non-robust automated IMPT plans with a standard five-field set-up was found in Supplementary Materials.

The median differences in D_50%_ for CTV_66/68Gy_ between automated and clinical IMPT plans were 0.2–0.3 Gy (Table 1) and were therefore not clinically relevant. For CTV_50Gy_, the median difference was 1 Gy lower and closer to the prescribed dose for automated than clinical plans, but the difference was only significant (*p* < 0.0001) for the automated plans with clinical field set-up. Experienced proton physicists mostly observed improved CTV_50Gy_ for automated plans at the sharp corners of the targets. However, CTV_60Gy_ coverage for automated plans was poorer than clinical plans.

**Table 1:** The median (1st quartile – 3rd quartile) dose of clinical target volumes (CTVs) for each of the four plan types: automated volumetric modulated arc therapy (VMAT) plan, automated intensity modulated proton therapy (IMPT) plan with a standard five-field set-up, automated IMPT plans with a clinical field set-up and clinical IMPT plans. D_50%_ is the dose received by 50% of the organ volume. V_95%_ is the organ volume that receive 95% of prescribed dose. CTV_68Gy_ and CTV_66Gy_ are the CTVs that were prescribed a dose of 68 Gy and 66 Gy, respectively. CTV_50Gy_ is the CTV that was prescribed a dose of 50 Gy, respectively, and then cropped against CTV_60Gy_.

	VMAT plan	Automated IMPT plan – five fields	Automated IMPT plan – clinical fields	Clinical IMPT plan
D_50%_ of CTV_68Gy_ (Gy)	68.1 (68.0–68.2)	68.4 (68.2–68.5)	68.3 (68.3–68.5)	68.0 (68.0–68.0)
D_50%_ of CTV_66Gy_ (Gy)	66.1 (66.0–66.1)	66.2 (66.1–66.2)	66.2 (66.1–66.3)	66.0 (66.0–66.0)
D_50%_ of CTV_50Gy_ (Gy)	50.0 (50.0–50.0)	50.5 (50.4–50.5)	50.5 (50.4–50.5)	51.5 (51.2–51.7)

**Fig. 2:**
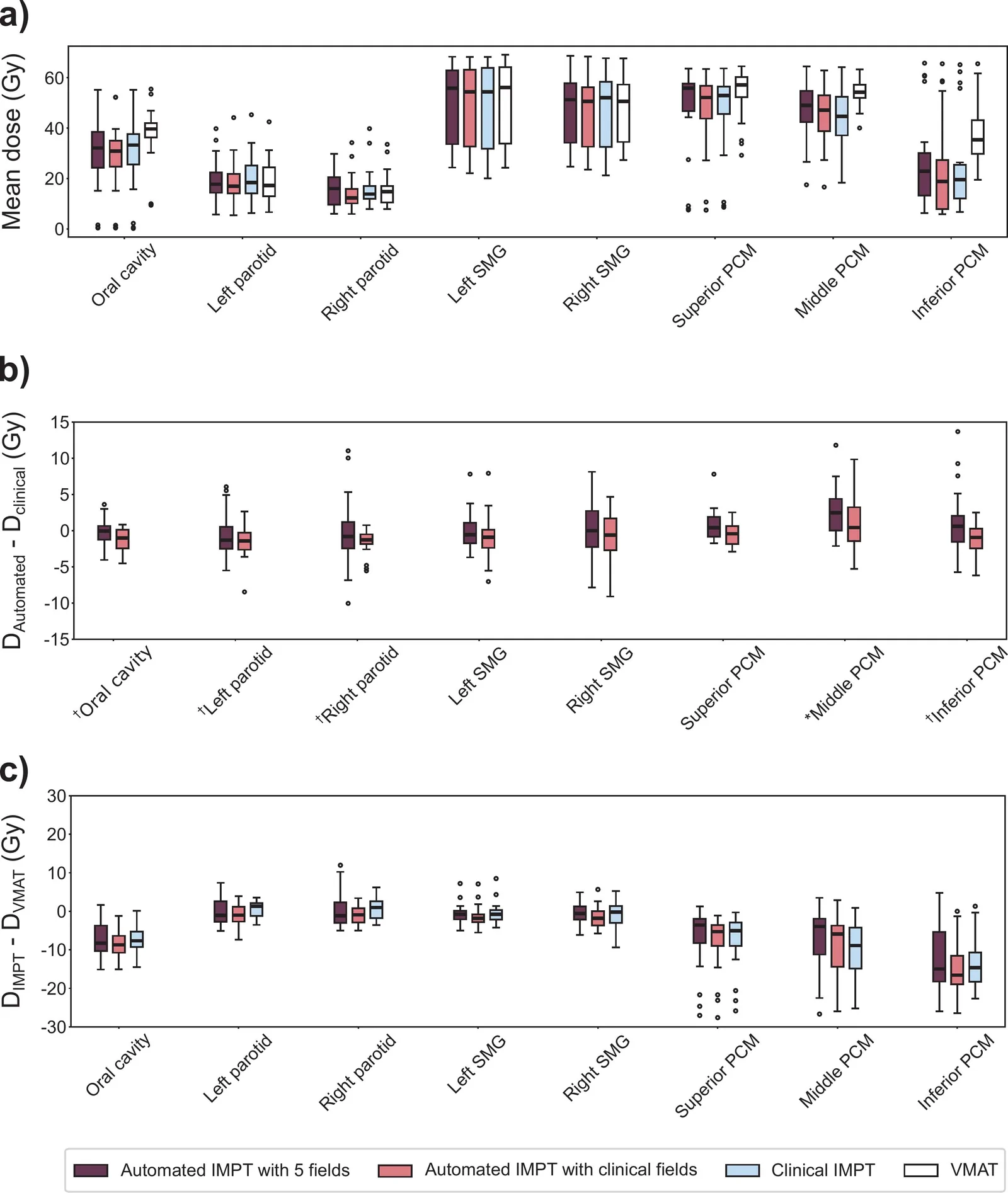
Box-whisker plots describing a) the mean dose, b) the difference in mean dose between automated and clinical intensity modulated proton therapy (IMPT) plans and c) the difference in mean dose between IMPT plans and volumetric modulated arc therapy (VMAT) plans for organs of interest used in the normal tissue complication probability model. The black line within the box, the top/bottom of the box and the whiskers indicate the median, the 75th/25th percentile and data laying within 1.5 times the inter-quartile-range from the box, respectively. * Statistically significant difference in mean dose between automated IMPT with 5 fields and clinical IMPT. † Statistically significant difference in mean dose between automated IMPT with clinical fields and clinical IMPT. SMG; Submandibular gland. PCM; pharynx constrictor muscle.

Fig. 2 shows boxplots of D_mean_ for OOIs included in the NTCP models for all plans (Fig. 2a), as well as the D_mean_ difference between automated and clinical IMPT plans (Fig. 2b), and between IMPT and VMAT plans (Fig. 2c). Automated optimisation mostly resulted in similar or reduced D_mean_ of OOIs. However, a median increase in D_mean_ for middle PCM of 2.4 Gy was observed for automated plans with a standard five-field set-up compared to clinical plans, although the difference was not significant. Automated plans with a clinical field set-up had significantly lower D_mean_ than clinical plans for left parotid (median difference = 1.4 Gy, *p*-value = 0.001) and right parotid (median difference = 1.3 Gy, p-value = 0.0001).

Compared with clinical IMPT plans, automated IMPT plan optimisation with both field set-ups significantly reduced D_max_ and D_mean_ for the spinal cord (p-value <0.0001), see Table 2. Automated optimisation with clinical fields also significantly reduced D_mean_ for the glottis (p-value <0.0001) and subglottis (p-value <0.0001). Table S.2 lists the *p*-values for all OOIs.

**Table 2:** The median (1st quartile – 3rd quartile) dose metric of organs of interest for each of the four plan types: volumetric modulated arc therapy (VMAT) plan, automated intensity modulated proton therapy (IMPT) plan with a standard five-field set-up, automated IMPT plan with a clinical field set-up and clinical IMPT plan. D_mean_ is the mean dose received by the organ, and D_max_ is the minimum dose given to the volume of 0.03 cm^3^ receiving the highest dose within the organ.

	VMAT plan (Gy)	Automated IMPT plan – five fields (Gy)	Automated IMPT plan – clinical fields (Gy)	Clinical IMPT plan (Gy)
Spinal cord, D_max_	39.4 (38.5–40.4)	19.9 (13.6–28.24)	16.9 (13.1–21.2)	35.9 (31.1–37.4)
Brainstem, D_max_	12.9 (5.3–27.0)	8.6 (1.8–15.8)	7.4 (1.6–10.1)	17.4 (1.7–24.6)
Spinal cord, D_mean_	19.1 (17.1–20.2)	3.0 (2.1–4.3)	3.1 (2.0–5.3)	5.9 (5.1–8.4)
Brainstem, D_mean_	2.1 (1.4–4.1)	0.4 (0.1–1.4)	0.4 (0.1–1.0)	0.5 (0.0–1.0)
Brain, D_mean_	0.8 (0.5–1.5)	0.2 (0.1–0.5)	0.1 (0.1–0.4)	0.1 (0.0–0.3)
Mandible, D_mean_	29.9 (24.4–34.8)	15.7(8.9–20.2)	14.7 (9.1–19.0)	17.8 (12.4–23.1)
Oesophagus, D_mean_	19.0 (13.8–23.7)	6.0 (3.7–8.1)	5.6 (3.4–7.8)	9.3 (4.7–12.5)
Glottis, D_mean_	28.6 (23.5–37.1)	10.5 (5.9–22.2)	9.1 (6.4–21.7)	13.7 (8.3–27.8)
Subglottis, D_mean_	50.7 (46.6–53.5)	40.2 (35.2–44.5)	39.7 (32.7–43.9)	41.8 (33.2–46.9)
Thyroid, D_mean_	36.1 (31.6–41.8)	33.7 (25.4–36.9)	28.9 (25.5–34.8)	30.4 (26.1–36.2)
Left buccal mucosa, D_mean_	33.8 (24.0–46.0)	5.6 (0.4–20.3)	5.0 (0.4–20.1)	4.9 (0.1–27.3)
Right buccal mucosa, D_mean_	24.9 (16.0–30.5)	1.6 (0.6–5.4)	1.3 (0.5–4.8)	0.8 (0.0–4.8)
Lips, D_mean_	8.9 (7.2–11.0)	0.3 (0.2–0.5)	0.3 (0.2–0.4)	0.0 (0.0–0.3)
Left hippocampus, D_mean_	1.2 (0.9–1.8)	0.1 (0.1–0.1)	0.1 (0.1–0.1)	0.0 (0.0–0-0)
Right hippocampus, D_mean_	1.1 (0.8–2.0)	0.1 (0.1–0.1)	0.1 (0.0–0.1)	0.0 (0.0–0-0)
Right cochlea, D_mean_	2.7 (1.9–5.6)	0.2 (0.1–0.3)	0.2 (0.1–0.3)	0.0 (0.0–0.1)
Left cochlea, D_mean_	2.8 (2.0–4.5)	0.3 (0.2–0.5)	0.3 (0.2–0.4)	0.0 (0.0–0.1)

**Fig. 3:**
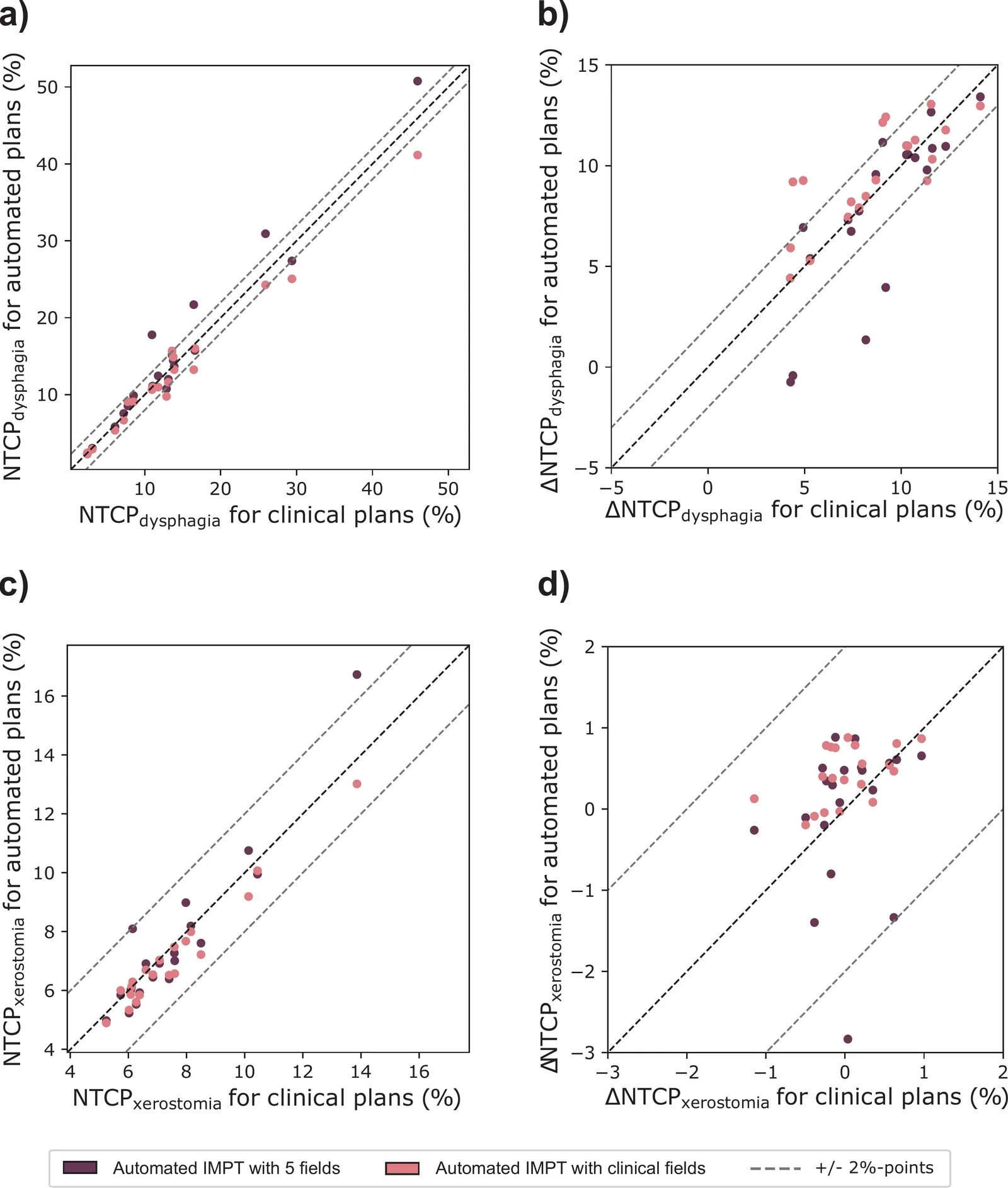
The normal tissue complication probability (NTCP) of automated intensity modulated proton therapy (IMPT) plans with either a standard five-field set-up or a clinical field set-up versus the NTCP of clinical IMPT plans for a) dysphagia and c) xerostomia. The ΔNTCP of automated IMPT plans plotted against ΔNTCP of clinical plans for b) dysphagia and d) xerostomia. ΔNTCP is the difference in NTCP between IMPT plans and volumetric modulated arc therapy plans (VMAT; VMAT-IMPT). The black dotted line is the identity line. The difference in NTCP or ΔNTCP between automated and clinical plans is less than 2%-points if the data point lies within the dotted grey lines.

Compared with clinical plans, the difference in NTCP and ΔNTCP of dysphagia for both types of automated IMPT plans was less than 2%-points for most patients (Fig. 3a and b). Four (20%) automated IMPT plans with a standard five-field set-up had 4.8–6.8%-points higher NTCP of dysphagia, while four (20%) automated IMPT plans with a clinical field set-up had 4.2–4.4%-points lower NTCP of dysphagia than clinical plans. However, no statistically significant NTCP differences were found between clinical plans and automated plans with either standard five-field or clinical field set-up. The difference in NTCP and ΔNTCP of xerostomia between automated and clinical IMPT plans were less than 2%-points for all but one plan (Fig. 3c and d).

## 4 Discussion

An efficient automated rule-based IMPT plan optimisation script for HNC was developed for a commercial TPS. It created robust plans with similar or lower OOI doses, resulting in similar or lower NTCP of dysphagia and xerostomia, than clinical plans for all patients when applying a clinical field set-up. With a standard five-field set-up, the script failed to achieve robust plans for some patients, leading to higher NTCP values than the clinical plans. The script created plans acceptable for comparative planning faster than manual planning, thereby saving time and resources.

Radiotherapy planning is associated with substantial inter-planner and inter-centre variability [18], [19], [20]. Automated plans may be considered clinically acceptable for comparative planning when they fall within this variability. NTCP differences in dysphagia and xerostomia between automated and clinical IMPT plans were less than 2%-points, excluding outliers. Studies evaluating inter-planner and inter-centre variability of dose distributions associated with manual HNC IMPT planning are scarce. Hansen et al. [21] compared NTCP for dysphagia and xerostomia for IMPT plans created at local Danish radiotherapy departments against clinical IMPT plans created at DCPT to evaluate whether local comparative planning was feasible. NTCP differences in the present study were smaller, especially for xerostomia, than differences reported in their study. This was expected, as their results were influenced by different CT scans and delineations. Nevertheless, it supports our conclusion that automated optimisation was adequate for comparative planning.

NTCP differences between automated and clinical IMPT plans may have resulted in different patient selection for those with NTCP close to the selection threshold. Automated IMPT plans with clinical field set-up had lower NTCP of dysphagia and xerostomia than the clinical plans for 75% of the patients. Huiskes et al. showed similar results, suggesting that automated optimisation may enhance plan quality [10]. Improved plan quality may reduce side effects and increase the likelihood that a patient is selected for IMPT.

In IMPT planning target volumes should be covered by at least two fields [15]. The standard five-field set-up failed to achieve this when there was minimal separation between the chin and shoulder. Clinical plans often used a posterior field to ensure robust target coverage under these conditions. Without a posterior field, automated optimisation failed to achieve robust CTV coverage, which limited the script's ability to reduce the dose of OOI objectives during optimisation. Consequently, automated optimisation with a standard five-field set-up resulted in substantially higher NTCP for four (20%) patients. The script has therefore been updated to add a posterior field when there is minimal separation between the chin and shoulder, allowing a standard field set-up to remain suitable for comparative planning. The updated script will be tested in the upcoming proton-versus-photon study for HNC in Norway (PRORADNOR).

Clinically, the “standard” field angles are often adjusted and lateral fields are added to improve CTV coverage and/or reduce OOI doses [22]. For plans that only required field angle adjustments and no additional fields, the NTCP difference between automated plans with a standard five-field and clinical field set-up was less than 1%-point, suggesting that field angle adjustments barely affected the NTCPs. However, D_mean_ of the middle PCM was higher for automated plans with a standard five-field set-up than clinical plans, likely due to additional lateral fields and/or field angle adjustments. This was supported by the lack of significant dose difference between automated plans with a clinical field set-up and clinical plans. Although additional lateral fields may reduce OOI dose, the NTCP was not substantially reduced when lateral fields were added to the standard field set-up. Hence, plans with standard five(six)-field set-up using an occasional posterior field can result in mean OOI doses and NTCP values similar enough to clinical plans for comparative planning.

Automated plans had significantly lower D_mean_ for multiple OOIs compared to the clinical plans. Although dose differences were significant, they had little clinical impact for the OOIs included in the NTCP models. However, larger dose differences were found for other OOIs. HNC treatment planning involves many OOIs, making optimisation complex. One advantage of the proposed automated optimisation is that it consistently attempts to reduce dose to all specified OOIs. In contrast to clinical optimisation, automated optimisation did not always restrict spot placement in cranial parts of the CTV for posterior and lateral posterior fields. This may have resulted in higher D_mean_ to hippocampus for automated than for clinical IMPT plans, but the difference in D_mean_ of 0.1 Gy is clinically irrelevant. Nevertheless, the updated script restricts the spot placement for posterior beams.

Different dose calculation matrices, spot placement approaches, TPSs, and dose calculation algorithms might also explain differences in dose and NTCP. While the automated IMPT plans with a clinical field set-up fulfilled the robust requirements for CTVs, proton physicists observed poorer nominal CTV_60Gy_ coverage for automated than for clinical IMPT plans. There is a natural trade-off between CTV coverage and OOI doses; thus, differences in CTV coverage may also explain the reduced OOI doses for automated compared with clinical plans. Despite different TPS and robustness, automated plans remain acceptable for comparative planning purposes.

The optimisation script reduced planning time from days to hours, enabling faster treatment decisions, which particularly benefit HNC patients. The optimisation script created IMPT plans in similar or shorter time than other rule-based optimisation methods [10], [13]. In contrast to knowledge-based optimisation [11], our optimisation script does not use training data and can easily be adapted to other fractionation regimes and tumour sites. Thus, the presented optimisation script is easy to implement in the clinic and may help initiate treatment earlier.

While the optimisation script created IMPT plans with comparable plan quality as clinical plans, it can be further developed to enhance plan quality. Manual field optimisation through trial and error was time-consuming and planner dependent. Although a patient-specific field set-up had little effect on the NTCP values in this study when disregarding the posterior field, Kong et al. showed that automated field optimisation can enhance plan quality and be achieved within an hour [13]. They also showed that increasing the number of fields reduced OOI doses, supporting that proton arc techniques may advance proton therapy [23]. It is possible to implement such advancements in the optimisation script.

The automated rule-based optimisation script efficiently produced robust IMPT plans with NTCPs comparable to clinical plans and is applicable for selecting HNC patients for IMPT. A standard five(six)-field set-up with an occasional posterior field is suitable for comparative planning for most patients.

## CRediT authorship contribution statement

**Marte Kåstad Høiskar:** Writing – review & editing, Writing – original draft, Visualization, Project administration, Methodology, Investigation, Formal analysis, Data curation, Conceptualization. **Ulrik Vindelev Elstrøm:** Writing – review & editing, Resources, Investigation, Data curation, Conceptualization. **Peter Sandegaard Skyt:** Writing – review & editing, Resources, Investigation, Data curation, Conceptualization. **Kathrine Røe Redalen:** Writing – review & editing, Supervision, Funding acquisition, Conceptualization. **Sigrun Saur Almberg:** Writing – review & editing, Writing – original draft, Visualization, Supervision, Methodology, Formal analysis, Conceptualization.

## Funding

This work was supported by NTNU Biotechnology.

## Declaration of competing interest

The authors declare that they have no known competing financial interests or personal relationships that could have appeared to influence the work reported in this paper.
