# Supplementary material for: Validation of automated script-based proton therapy optimisation for comparative head and neck planning
Source: Phys Imaging Radiat Oncol. 2026 Aug 5;40:101058. doi: 10.1016/j.phro.2026.101058 (PMC13495582; doi:10.1016/j.phro.2026.101058)
Supplement: Supplementary file 1 [file mmc1.pdf]

Validation of Automated Script-based Proton Therapy Optimisation for Comparative Head and Neck Planning

Supplementary Materials

Marte Kåstad Høiskar[a], Ulrik Vindelev Elstrøm[b], Peter Sandegaard Skyt[b], Kathrine Røe Redalen[a], Sigrun Saur Almberg[c]

[a]Department of Physics, Norwegian University of Science and Technology, Trondheim, Norway

[b]Danish Centre for Particle Therapy, Aarhus University Hospital, Denmark

[c]Department of Radiotherapy, Cancer Clinic, St. Olavs Hospital, Trondheim University Hospital, Trondheim, Norway

CTV coverage of non-robust automated IMPT plans

Some of the automated IMPT plans with a standard five-field set-up did not achieve robust CTV coverage with $V_{95\%}$ of >98%, >98% and >99% for CTV_{50Gy}, CTV_{60Gy} and $CTV_{66/68Gy}$, respectively. Table S.1 describes the $V_{95\%}$ of CTV_{50Gy}, CTV_{60Gy} and $CTV_{66/68Gy}$ for the worst-case scenario of these automated IMPT plans, together with number of passed scenarios out of 14 scenarios. Patient 4 had the worst coverage for all CTVs. The shoulder and chin of this patient overlapped with 3 cm in the caudal-cranial direction where none of the fields in the five-field configuration were allowed to contribute. This explains the poor target coverage, which may be improved with a posterior field (180-degree). Similar overlap was seen for other patients as well, but not to this extent.

Table S.1: The $V_{95\%}$ of CTV_{50Gy}, CTV_{60Gy} and $CTV_{66/68Gy}$ for the worst-case scenario of automated IMPT plans with a standard five-field set-up that did not fulfil the robust CTV (clinical target volume) coverage criteria. V_{95} refers to the organ volume receiving 95% of the prescribed dose. CTV_{50Gy}, CTV_{60Gy} and $CTV_{66/68Gy}$ are the CTVs that were prescribed 50 Gy, 60 Gy and 66/68 Gy, respectively. The $V_{95\%}$ of CTV_{50Gy}, CTV_{60Gy} and $CTV_{66/68Gy}$ that did not fulfil the criteria are marked in red, while those that did are marked in green. The number of scenarios out of 14 evaluated scenarios that passed the criteria are also described.

Patient	CTV_{50Gy} (passed scenarios)	CTV_{60Gy} (passed scenarios)	$CTV_{66/68Gy}$ (passed scenarios)
1	> 98% (14)	97.9% (13)	> 99% (14)
2	> 98% (14)	> 98% (14)	98.9% (13)
3	> 98% (14)	> 98% (14)	99.0% (13)
4	96.3% (0)	97.5% (13)	98.3% (13)
5	> 98% (14)	97.7% (13)	97.2% (12)
6	> 98% (14)	> 98% (14)	98.5% (12)

Table S.2: The p-value of the Wilcoxon signed rank test, testing the null hypothesis that the difference in D_{mean} or D_{max} between volumetric modulated arc therapy (VMAT) plans or automated intensity modulated proton therapy (IMPT) plans and clinical IMPT plans is zero for organs of interest. D_{mean} is the mean dose, while D_{max} is the minimum dose given to the volume of 0.03 cm^3 receiving the highest dose within the organ.

	VMAT plan (Gy)	Automated IMPT plan – five fields (Gy)	Automated IMPT plan – clinical fields (Gy)
Spinal cord, D_{max}	0.0003	<0.0001	<0.0001
Brainstem, D_{max}	0.04	0.07	0.0012
Brain, D_{max}	0.07	0.017	0.0012
Spinal cord, D_{mean}	<0.0001	<0.0001	<0.0001
Brainstem, D_{mean}	<0.0001	0.033	0.14
Brain, D_{mean}	<0.0001	0.0002	0.0032
Mandible, D_{mean}	<0.0001	0.0064	<0.0001
Oesophagus, D_{mean}	<0.0001	0.027	<0.0001
Glottis, D_{mean}	<0.0001	0.23	<0.0001
Subglottis, D_{mean}	<0.0001	0.08	<0.0001
Thyroid, D_{mean}	<0.0001	0.84	0.02
Left buccal mucosa, D_{mean}	<0.0001	0.76	0.09
Right buccal mucosa, D_{mean}	<0.0001	0.96	0.67
Lips, D_{mean}	<0.0001	0.12	0.20
Left hippocampus, D_{mean}	0.004	0.004	0.004
Right hippocampus, D_{mean}	0.004	0.004	0.004
Right cochlea, D_{mean}	<0.0001	0.096	0.09
Left cochlea, D_{mean}	<0.0001	0.08	0.08
Oral cavity, D_{mean}	<0.0001	0.65	0.005
Superior PCM, D_{mean}	<0.0001	0.07	0.26
Middle PCM, D_{mean}	<0.0001	0.004	0.50
Inferior PCM, D_{mean}	<0.0001	0.3	0.03
Right parotid, D_{mean}	0.50	0.57	<0.0001
Left parotid, D_{mean}	0.30	0.29	0.0014
Right submandibular gland, D_{mean}	0.40	0.81	0.45
Left submandibular gland, D_{mean}	0.09	0.5	0.11
